# Computer-aided detection (CAD) system for breast MRI in assessment of local tumor extent, nodal status, and multifocality of invasive breast cancers: preliminary study

**DOI:** 10.1186/s40644-015-0036-2

**Published:** 2015-02-08

**Authors:** Sung Eun Song, Bo Kyoung Seo, Kyu Ran Cho, Ok Hee Woo, Gil Soo Son, Chulhan Kim, Sung Bum Cho, Soon-Sun Kwon

**Affiliations:** Department of Radiology, Korea University Ansan Hospital, Korea University College of Medicine, 123 Jeokgeum-ro, Danwon-gu, Ansan city, Gyeonggi-do 425-707 Korea; Department of Radiology, Korea University Anam Hospital, Korea University College of Medicine, 73 Inchon-ro, Seongbuk-gu, Seoul, 136-705 Korea; Department of Radiology, Korea University Guro Hospital, Korea University College of Medicine, 148 Gurodong-ro, Guro-gu, Seoul, 152-703 Korea; Department of Surgery, Korea University Ansan Hospital, Korea University College of Medicine, 123 Jeokgeum-ro, Danwon-gu, Ansan city, Gyeonggi-do 425-707 Korea; Department of Nuclear Medicine, Korea University Ansan Hospital, Korea University College of Medicine, 123 Jeokgeum-ro, Danwon-gu, Ansan city, Gyeonggi-do 425-707 Korea; Department of Biostatistics, Seoul National University Bundang Hospital, 82 Gumi-ro, 173 Beon-gil, Seongnam-si, Gyeonggi-do 463-707 Korea

**Keywords:** Breast, Breast neoplasm, Tumor staging, Computer-assisted diagnosis, Magnetic resonance imaging

## Abstract

**Background:**

We aimed to investigate the efficacy of computer-aided detection (CAD) for MRI in the assessment of tumor extent, lymph node status, and multifocality in invasive breast cancers in comparison with other breast imaging modalities.

**Methods:**

Two radiologists measured the maximum tumor size, as well as, analyzed lymph node status and multifocality in 86 patients with invasive breast cancers using mammography, ultrasound, CT, MRI with and without CAD, and 18-fludeoxyglucose positron emission tomography (FDG-PET). The assessed data were compared with pathology.

**Results:**

For tumor extent, there were no significant differences between pathological size and measured size using mammography, ultrasound, CT, or MRI with and without CAD (*P >* 0.05). For evaluation of lymph node status, ultrasound had the best kappa coefficients (0.522) for agreement between imaging and pathology, and diagnostic performance with 92.1% specificity and 90.0% positive predictive value. For multifocality, MRI with CAD had the highest area under the receiver operating characteristic curve (AUC = 0.888).

**Conclusions:**

CAD for MRI is feasible to assess tumor extent and multifocality in invasive breast cancer patients. However, CAD is not effective in evaluation of nodal status.

## Background

Precise information regarding tumor extent, lymph node (LN) status, and multifocality is of great importance in breast cancer treatment and prognosis, as well as, recurrence prediction. In particular, negative margins in breast-conserving therapy significantly affect local tumor recurrence [1,2]. Axillary LN status is also a major prognostic indicator and acts as a guide, in terms of the need for adjuvant chemotherapy, in breast-cancer patients [3]. Multimodal breast imaging techniques for the preoperative assessment of breast cancer staging are available, especially mammography (MMG), ultrasound (US), computed tomography (CT), magnetic resonance imaging (MRI), and 18-fludeoxyglucose positron emission tomography (FDG-PET). There are several studies comparing the diagnostic performance of these modalities, with breast MRI reported to be the most accurate imaging modality for assessment of tumor extent and LN status [4-7].

Breast MRI, however, has various specificity, and high false positive rates, when detecting breast cancers and also tends to overestimate tumor extent [8-11]. Thus, physicians have not achieved a consensus on the utilization of MRI staging prior to surgery. Evaluation of breast cancer using MRI takes significant time for image processing and interpretation [12,13]. In addition, inter- and intra-observer variations are an additional drawback of breast MRI [14].

Various commercially available computer-aided detection (CAD) systems for breast MRI have been introduced to address these limitations. CAD automates and speeds up image processing and analysis functions [10], and detects breast lesions by using an enhancement threshold. The system provides both morphologic and kinetic features of the breast lesion, and also provides quantitative information, such as, lesion dimensions and maximum tumor volumes. Levrini et al. [15] reported that CAD is just as accurate as breast MRI for size assessment of breast lesions. In addition, Bhooshan et al. [6] demonstrated that CAD for MRI could differentiate between invasive and noninvasive breast lesions, and also, between invasive breast cancers with LN metastasis and invasive cancers without LN metastasis. There are few clinical reports, however, showing the utility of CAD for MRI in tumor staging [6,15].

The purpose of this preliminary study was to compare diagnostic accuracy for preoperative assessment of tumor extent, LN status, and multifocality in invasive breast cancers among multimodal breast imaging options, such as, MMG, US, CT, MRI, and FDG-PET. In addition, our specific goal was to investigate if CAD for MRI can be extended from cancer detection to cancer staging for the determination of treatment and prognosis in clinical settings.

## Methods

### Patient population

Institutional review board approval was obtained from the Korea University Ansan Hospital, and the requirement for informed consent was waived for this retrospective review. We searched our institution’s database for invasive breast cancer patients who underwent all imaging modalities from August 2008 to February 2012, including MMG, US, CT, MRI with CAD, and FDG-PET before breast cancer surgery. Of 211 invasive breast cancer patients, 147 patients, who underwent all imaging modalities, were initially included. Among these 147 patients, we excluded 34 patients who underwent neoadjuvant chemotherapy, or excisional biopsy, within 6 months because recent neoadjuvant chemotherapy, or excisional biopsy, may produce complications or changes in surrounding breast tissues, which could interfere with the pathologic-radiologic correlation. Further, of the 34 exclusions, twenty patients of the patients had no surgical confirmation, and seven patients did not receive sentinel LN biopsy or axillary LN dissection. Four patients who had no detectable lesions on CAD were included in our study to reflect possible false negative results of CAD. Therefore, a total of 86 patients (mean ages: 49.18, range: 28–82 years old) were included in this study. Pathological diagnoses of these patients included 77 invasive ductal carcinomas, four invasive micropapillary carcinomas, one invasive lobular carcinoma, one invasive apocrine carcinoma, one invasive mucinous carcinoma, one invasive cribriform carcinoma, and one adenoid cystic carcinoma. Forty-five patients underwent modified radical mastectomy, and the remaining 41 patients underwent breast conserving surgery. All patients underwent sentinel LN biopsy, and 56 patients underwent axillary LN dissection.

### Imaging techniques

All 86 patients underwent multimodal breast imaging using a digital MMG unit (Selenia FFDM system, Hologic Cooperation, Denver, CO, USA), US units (LOGIQ 9 unit, GE Medical Systems, Milwaukee, Wis, USA and iU22, Philips Medical Systems, Bothell, WA, USA), a 64-channel multidetector CT scanner (Brilliance 64-channel, Philips Medical Systems, Cleveland, OH, USA), a 3 T MRI system (Achieva 3.0 T TX, Philips Medical Systems, Best, the Netherlands), and a PET/CT scanner (Gemini TF/Brilliance 16, Philips Medical Systems, Best, the Netherlands).

MMG captured both craniocaudal and mediolateral oblique views. US examination was performed in bilateral whole breasts, and axillae, with high-frequency linear transducers, also, survey scanning was performed in transverse and sagittal planes. CT examinations were performed by dynamic study after intravenous infusion of 130 ml of nonionic contrast media (Iopamiro 300, Bracco, Italy or Ultravist, Schering, Germany) at a rate of 2.0 ml/sec. Early phase post-contrast images were obtained 90 seconds after contrast media injection, and delayed phase post-contrast images at 300 seconds after injection. The CT scan was performed at a low-dose setting (120 kVp and 30 or 50 mAs).

For MRI, we obtained axial T2-weighted turbo spin echo images, dynamic sagittal T1-weighted gradient echo images with fat saturation, and axial and coronal 3D reconstruction images with maximum intensity projection. T2-weighted scan was performed; TE/TR 120/9022 ms, inversion delay spectral attenuated inversion recovery (SPAIR), 125 ms, flip angle 90°, field of view (FOV) 340 × 340 mm^2^, acquired voxel size 1.01 × 1.31 × 2.0 mm^3^, reconstructed voxel size 0.66 × 0.66 × 2.00 mm^3^. Fat-saturated T1-weighted images were acquired before and after contrast media injection with a total of six dynamic acquisitions; TE/TR 1.3/3.4 ms, flip angle 10°, FOV 320 × 320 mm^2^, acquired voxel size 0.91 × 0.91 × 2.00 mm^3^, reconstructed voxel size 0.83 × 0.83 × 1.00 mm^3^. Twenty ml of 0.5 mmol/ml Gadodiamide (Omniscan, Nycomed-Amersham, Princeton, NJ, USA) was injected intravenously followed by a 20-ml saline flush at the rate of 2.0 ml/sec. All collected MRI data were processed using a commercially available CAD system (CADstream software, version 5.2.8.591, Merge Healthcare, Chicago, IL, USA).

For FDG-PET scan, CT images were obtained after FDG injection and then PET scans were performed. PET acquisition data were reconstructed using the 3D row action maximum likelihood algorithm. Reconstruction was performed to yield slices containing 144 × 144 pixels. We obtained 3D maximum intensity projection images, axial images with a slice thickness of 4 mm, and coronal and sagittal images with a slice thickness of 4.6 mm.

### Image evaluation

Two radiologists with 3 or 13 years of experience in breast imaging interpreted all images using a picture archiving and communication system (PACS). One radiologist had 3 years of experience in CAD reading and the other had 5 years of experience. The radiologists used consensus double reading. Each reader independently evaluated images and the final decision was reached by discussion between the two readers. US images and mammograms were reviewed at the time of CT, MRI, or FDG-PET interpretation. The radiologists were blinded to the final pathological results. The Breast Imaging Reporting and Data System (BI-RADS) lexicon was used for diagnosis of malignant tumor on MMG, US, and MRI [16]. Morphology on MMG, US, and MRI, and kinetic features on MRI were evaluated. On breast CT, focally-enhancing lesions, after contrast injection, were evaluated [17]. On PET/CT, breast lesions were suspected to be malignant if they showed elevated tracer uptake when compared with the adjacent breast tissue.

**Figure 1:**
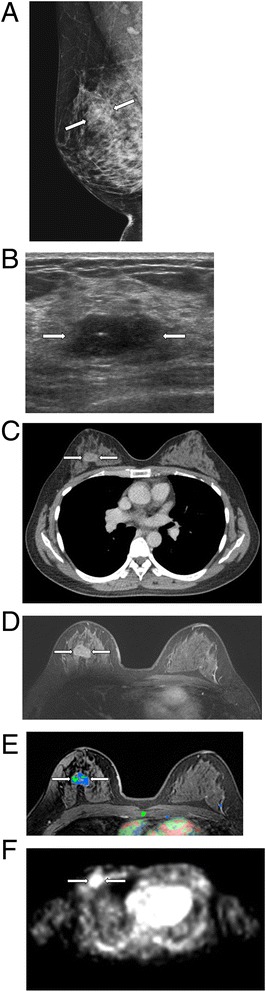
**How to measure the tumor extent.** The longest diameter of a breast tumor was recorded using each modality; MMG **(A)**, US **(B)**, CT **(C)**, MRI without CAD **(D)**, MRI with CAD **(E)**, and FDG-PET **(F)**. For measurement of tumor extent with CAD, an enhancing tumor above the threshold was selected with the color overlay map, and then CAD-generated maximum tumor size was recorded.

For tumor extent, the longest diameter of a breast tumor was recorded using each modality (Figure 1). On MMG, tumor sizes were measured with both craniocaudal and mediolateral oblique views, and then the maximum length was chosen. Tumor sizes were measured on multi-sections using US, CT, FDG-PET, and MRI, and then the longest length was selected. CAD system automatically provided maximum tumor extent if we clicked the enhancing tumor.

A lymph node was considered as metastasis if it had more than one of these characteristics: 1) the longitudinal-transverse axis ratio of the LN was less than two; 2) the cortex of the LN was concentrically or eccentrically thickened more than 3 mm; 3) fatty hilum was absent or displaced; 4) there was extranodal fat infiltration; 5) the LN showed early enhancement with a delayed washout pattern on dynamic CT and MRI; or 6) the LN showed increased standardized uptake value (SUV) on FDG-PET. If the axillary LN exhibited any of the findings mentioned above, it was defined as metastatic LN. LNs that exhibited none of the above mentioned criterion were defined as negative for axillary LN metastasis [18,19]. On MRI, we used fat-saturated T1-weighted imaging for assessment of LN status, and we often used precontrastT2-weighted images for evaluation of extranodal fat infiltration. We also evaluated presence of multifocality of breast carcinomas on all imaging modalities.

For CAD analysis, a color overlay map was placed on all enhancing lesions at a 50% enhancement threshold level in a pixel by pixel comparison across a pre-contrast, early post-contrast, and delayed post-contrast series, and set threshold of 50% could not be altered during the analysis of individual lesions. Numerous prior studies have used higher enhancement thresholds, more than 50%, to improve specificity [13,20,21]. Levman et al. [22]. insisted that enhancement thresholds can limit a CAD test’s ability to diagnose a lesion’s full size and, as such, should not be raised above 60%. However, the signal enhancement ratio (SER) method exhibits a high rate of false positives at low enhancement thresholds. Therefore, they demonstrated that the most appropriate threshold would be 50%–60%. Based on these previous studies, we selected a 50% threshold in this study.

The initial phase determined by the signal change between the pre-contrast and early, post-contrast, series was classified as medium (50–100%), or rapid (>100%) enhancement. Delayed-phase enhancement type, after early post-contrast series, was classified as persistent, plateau, or washout. The washout type, displayed in red, represented pixel signal intensity with at least a 10% decrease in the delayed post-contrast series compared to the early post-contrast series. The persistent type, displayed in blue, represented pixel signal intensity with at least a 10% increase in the delayed post-contrast series compared to the early post-contrast series. The plateau type, displayed in green, represented pixel signal intensity with a less than 10% increase, and a less than 10% decrease, compared to the early post-contrast series. Morphologic evaluation was also performed based on the BI-RADS lexicon [16]. Although a lesion had morphologically non-malignant features, such as, a circumscribed margin and oval shape, we considered it as malignant if the lesion had malignant kinetic features or strong enhancement about the threshold on CAD. For measurement of tumor extent with CAD, an enhancing tumor above the threshold was selected with the color overlay map, and then the, CAD-generated, maximum tumor size was recorded. For evaluation of nodal status on CAD, LNs above threshold enhancement were individually selected by radiologists and the presence of significant enhancement, as indicated by the presence of a color overlay map on the LN, was recorded as positive LN. These findings were confirmed using same-level breast MRI images because CAD sometimes detected engorged axilla vessels as positive LN. We determined positive or negative LN based on morphologic and kinetic features on CAD.

### Pathological examination

Pathologic specimens were obtained within 2 weeks after the acquisition of preoperative images. Specimens were prepared by making serial 5-mm slices of breast-conserving surgical specimens, and 5–10-mm slices of mastectomy specimens, according to the technique reported by Egan [23]. For the correlation of pathological findings and breast imaging features, pathologists and radiologists reviewed all specimens together. For assessment of LN status, sentinel lymph node biopsy was performed and then evaluated with frozen microscopic examinations. If there was a positive sentinel lymph node, axillary node dissection was performed. For diagnosis of multifocality, an imaging-guided core biopsy, prior to surgery, or a needle-localization and excisional biopsy, during surgery, was performed on suspicious additional breast lesions. Pathologists reviewed all pathologic findings and reported tumor extent, nodal status, and multifocality of invasive breast carcinomas.

### Analysis

Evaluated tumor extent, LN status, and multifocality of invasive breast carcinomas on breast-imaging modalities were compared with actual pathological results. For assessment of the accuracy of tumor extent on each modality, correlation coefficients were calculated using the Spearman rank correlation test in order to assess the strength of the linear association between the pathologic tumor size, and the recorded size, from each modality. The pathologic tumor size was considered to be the tumor extent on pathological examination. The Mann–Whitney test was used to investigate whether there were size differences between the pathologic tumor size and the recorded size on each imaging modality. Further, the Kruskal-Wallis test was used to study whether there were significant differences between various imaging modalities for the assessment of tumor extent.

Weighted kappa coefficients were used to find the agreement of LN status between multimodal breast imaging and actual pathological results. A kappa coefficient greater than 0.80 indicated excellent agreement, 0.61-0.80 good agreement, 0.41-0.60 moderate agreement, 0.21-0.40 fair agreement, and less than 0.20 poor agreement [24]. The chi-square test was used to compare the LN status on multimodality breast imaging and final pathological results. Sensitivity, specificity, positive predictive value (PPV), and negative predictive value (NPV) were calculated.

For comparison of the accuracies of diagnosis of multifocality on the various imaging modalities, standard receiver operating characteristic analysis was used to generate sensitivity, specificity, and area, under the ROC curve (AUC) of imaging modalities used for detecting multifocality in a breast cancer patients. AUC equal to 1 indicated a perfect test, 0.7-0.9 moderate accuracy, 0.5-0.7 less accuracy, and equal to 0.5 was non-informative. Statistical analyses were performed using SAS version 9.1 (SAS Institute, Cary, NC, USA). P values equal to or less than 0.05 were accepted as statistically significant.

## Results

### Tumor extent

Table 1 demonstrates lesion characteristics of all 82 invasive carcinomas with measured tumor extent on multimodal breast imaging including CAD. Mean and median values of tumor extent, and correlation coefficients between pathologic tumor size and recorded size on multimodal breast imaging, are shown on Table 2. The median pathological tumor extent was 22 mm. All imaging modalities yielded statistically significant measurements with a range of correlation coefficients, 0.513-0.766 (*P* < 0.05). The correlation coefficient was highest in MRI without CAD, followed by CT, US, MMG, FDG-PET, and MRI with CAD. The Kruskal-Wallis test was used to investigate whether there were statistically significant size differences between all imaging modalities, and the result indicated there were no significant size differences between the six imaging types (*P* = 0.165). In addition, the Mann–Whitney test was used to investigate if there were differences between pathologic tumor size and recorded tumor size. There were no significant differences between pathologic tumor size and recorded size on US, CT, or MRI with or without CAD (*P >* 0.05), but there was a significant size difference between pathologic tumor size and recorded size on FDG-PET (*P* = 0.007).

**Table 1:** **Lesion characteristics of 86 invasive breast carcinomas using multimodal breast imaging**

**Imaging modality**	**Lesion characteristics**
Mammography	Mass 29 (33.6%)
	Calcifications 3 (3.5%)
	Mass with calcifications 36 (41.9%)
	Asymmetry 18 (21.0%)
Ultrasound	Mass 46 (53.5%)
	Mass with calcifications 32 (37.2%)
	Mass with abnormal ducts 8 (9.7%)
CT	Enhancing mass 86 (100.0%)
MRI	Mass 76 (88.4%)
	Non-mass-like enhancement 10 (11.6%)
FDG-PET	Elevated tracer uptake with enhancing mass 86 (100.0%)

*CT* computed tomography, *MRI* magnetic resonance imaging, *FDG-PET* 18-fludeoxyglucose positron emission tomography.

**Table 2:** **Tumor size and correlation coefficients representing the strength of a linear association between pathological tumor sizes and recorded sizes with multimodal breast imaging**

	**Pathology**	**Mammography**	**Ultrasound**	**CT**	**MRI without CAD system**	**MRI with CAD system**	**FDG-PET**
Mean tumor size	25.2 ± 14.8	30.7 ± 19.1	28.5 ± 17.7	27.6 ± 15.6	29.1 ± 16.3	28.3 ± 23.1	32.7 ± 21.5
Median tumor size	22	27	23	22	24	22	28
Size Range	(3–74)	(9–120)	(5–100)	(8–79)	(8–78)	(0.5-100)	(0–103)
Correlation coefficients		0.583	0.656	0.711	0.766	0.513	0.616

*CT* computed tomography, *MRI* magnetic resonance imaging, *CAD* computer-aided detection system, *FDG-PET* 18-fludeoxyglucose positron emission tomography.

### LN status

Table 3 demonstrates weighted kappa coefficients for agreement of LN status between multimodality breast imaging and actual pathological results. The coefficients ranged from 0.158 to 0.420. US and CT had moderate agreement. MMG, MRI without CAD, and FDG-PET had fair agreement. CAD for MRI showed the worst result; poor agreement.

**Table 3:** **Weighted kappa coefficients for agreement of LN status between multimodality breast imaging and pathological results**

	**Mammography**	**Ultrasound**	**CT**	**MRI without CAD system**	**MRI with CAD system**	**FDG-PET**
Weighted kappa coefficients	0.223	0.522	0.420	0.346	0.158	0.285

*CT* computed tomography, *MRI* magnetic resonance imaging, *CAD* computer-aided detection system, *FDG-PET* 18-fludeoxyglucose positron emission tomography.

Table 4 shows diagnostic performance of LN status with multimodal breast imaging assessed using the Chi-square test. All imaging modalities had higher values of specificity and PPV than those of sensitivity and NPV. US was the best imaging modality, with higher values of sensitivity (61.4%), specificity (92.1%), PPV (90.0%), and NPV (67.3%). MMG and FDG-PET had high values of specificity (86.8%, 81.6% respectively) and PPV (76.2%, 75.0%), however, sensitivity (36.4%, 47.7%) and NPV (54.1%, 57.4%) were very low. CT and MRI without CAD had a range of 61.4%-78.9% in all diagnostic performance parameters. CAD for MRI did not improve diagnostic performance when compared with MRI without CAD.

**Table 4:** **Diagnostic performance of the determination of LN status with multimodality breast imaging**

	**Mammography**	**Ultrasound**	**CT**	**MRI without CAD system**	**MRI with CAD system**	**FDG-PET**
Sensitivity (%)	36.4	61.4	63.6	61.4	47.7	47.7
Specificity (%)	86.8	92.1	78.9	73.7	68.4	81.6
PPV (%)	76.2	90.0	77.8	73.0	63.6	75.0
NPV (%)	54.1	67.3	65.2	62.2	53.1	57.4

*CT* computed tomography, *MRI* magnetic resonance imaging, *CAD* computer-aided detection system, *FDG-PET* 18-fludeoxyglucose positron emission tomography, *PPV* positive predictive value, *NPV* negative predictive value.

### Multifocality

**Figure 2:**
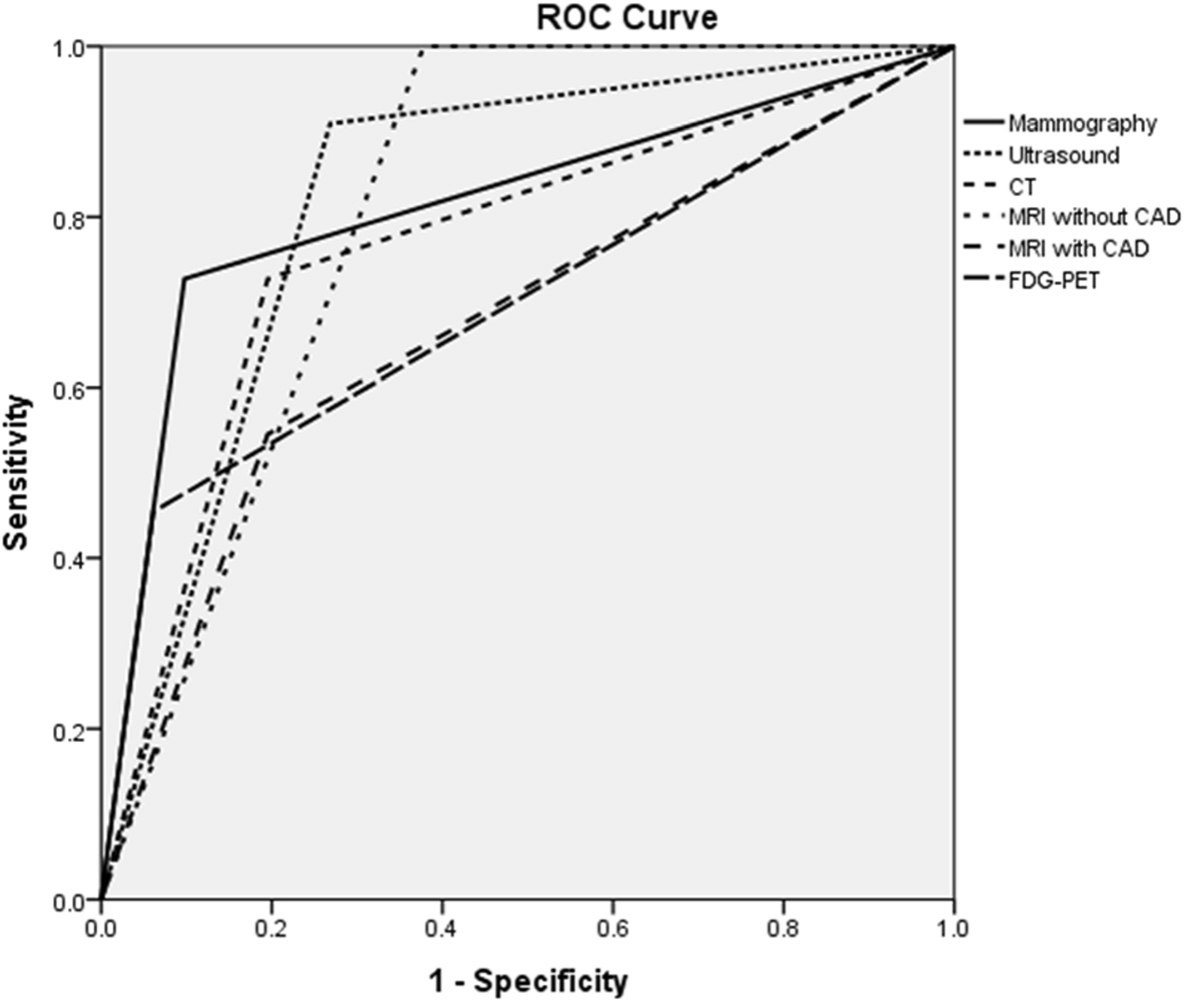
**Summary of ROC curve of each imaging modality for detecting multifocality in breast cancer patients.** AUC was 0.781 for MMG, 0.772 for US, 0.728 for CT, 0.809 for MRI without CAD, 0.888 for MRI with CAD and 0.634 for FDG-PET.

Six of the total 86 patients had multiple carcinomas on pathologic examination. The number of multifocal lesions for each patient ranged from two to four. Five of these six (83.3%) patients underwent mastectomy and only one patient underwent breast conserving surgery. The surgical plan was changed in three (50%) patients due to multifocality. Table 5 and Figure 2 present the sensitivity, specificity and AUC of each imaging modality for detecting multifocality in breast cancer patients. Sensitivity was the best in MRI with or without CAD (100%, 6/6) compared with other imaging modalities. FDG-PET had the highest value of specificity (93.4%, 71/76), however, it had the lowest value of sensitivity (33.3%, 2/6). AUC ranged from 0.634 to 0.888. MRI with CAD had the highest AUC value. All imaging modalities except FDG-PET yielded statistically significant AUC and were moderately accurate (0.7 < AUC < 0.9) at detecting multifocality. Sensitivities of MRI with or without CAD for detecting multifocality of breast cancer were 100%. MRI with CAD had superior specificity (77.6%) when compared with that of MRI without CAD (61.8%).

**Table 5:** **Overall sensitivity, specificity, and AUC for evaluation of multifocality with multimodality breast imaging**

	**Mammography**	**Ultrasound**	**CT**	**MRI without CAD system**	**MRI with CAD system**	**FDG-PET**
Sensitivity (%)	66.7	83.3	66.7	100.0	100.0	33.3
Specificity (%)	89.5	71.1	79.0	61.8	77.6	93.4
AUC	0.781	0.772	0.728	0.809	0.888	0.634
*P* value	0.00	0.00	0.03	0.00	0.00	0.20

*CT* computed tomography, *MRI* magnetic resonance imaging, *CAD* computer-aided detection system, *FDG-PET* 18-fludeoxyglucose positron emission tomography, *PPV* positive predictive value, *NPV* negative predictive value, *AUC* area under the ROC curve.

## Discussion

Breast cancers are dependent on angiogenesis for growth and development, because they need blood vessels to obtain nutrients [25]. Dynamic breast MRI is sensitive to variations in vascular permeability and blood volume, which can be associated with tumor angiogenesis [26]. Signal enhancement ratio, a quantitative method for characterizing angiogenesis in breast cancer, measures changes in contrast signal intensity over three time points and acts as a surrogate marker for contrast kinetics [27]. CAD uses the SER to compute the angiomap which refers to an image with color-coded tumors representing kinetic information and provide volume, maximal intensity projection, multi-planar reform, and the curve classification of enhancing lesions. As suggested by Hylton [25], SER can provide signs that correlate with angiogenesis. In detail, CAD incorporates unenhanced images and contrast-enhanced images and then compares pixel signal intensity values on the immediate- and delayed-contrast enhanced images to indicate washout, plateau, or persistent enhancement pattern based on pixel value increases above a user-specified minimum enhancement threshold [28]. Specific colors such as blue, green and red are assigned to each pixel for different types of tissue enhancement, such as persistent, plateau and washout, respectively [28]. CAD is a post-processing software program that performs image analysis and integrates with MRI automatically. Its abilities to promptly analyze considerable numbers of images, facilitate reconstructions, aid in visual subtraction, and increase tumor detection rates are considerable advantages. CAD also improves the interpretation efficiency and diagnostic performance of radiologists by reducing reading time and maintaining the consistency of lesion detection [12].

CAD for MRI has been developed for breast cancer detection. Although the system provides automatic size and volume data and regional LN information, up to now there have been few reports that have demonstrated the utility of CAD for preoperative tumor staging [6,15]. In this study, we investigated the feasibility of CAD for assessment of tumor extent, LN status, and multifocality in invasive breast cancers by comparing CAD for MRI with other breast imaging modalities, including MMG, US, CT, MRI without CAD, and FDG-PET.

In terms of tumor extent, all imaging modalities tended to overestimate tumor size compared with actual pathological size, but there was no significant difference between recorded size and pathological size, except with FDG-PET. Small underestimation of actual pathological size can be explained by shrinkage of tissues during fixation and processing [29]. On FDG-PET, breast cancers with higher SUV can be overestimated due to the overflow effect and small cancers with lower SUV can be underestimated due to the partial volume effect. In addition, size measurement is affected by manipulation of scale bar. Uematsu et al. [7] and Heusner et al. [30] also reported that FDG-PET has significantly lower accuracy than MRI for measurement of tumor extent and, thus, is not to be recommended for this purpose. In our study, manual measurement on MRI was the best modality to assess tumor extent. US, CT, and MRI were more accurate than mammography, and thus, good imaging types to assess breast cancer staging. Although the correlation coefficient of MRI without CAD was higher than that of MRI with CAD, there was no statistically significant difference between automatically measured tumor size on CAD and manually measured tumor size on MRI in this study. However, a preliminary study by Demartini et al. [31] with 15 patients demonstrated CAD tumor sizes are less accurate in predicting the size of residual malignancy than those measured by the radiologist. Therefore, further study in a large population would be necessary to evaluate utility of automatic tumor measurement of CAD.

In terms of LN status, MMG, US, CT, MRI, and FDG-PET all had fair-to-moderate agreement between breast imaging and actual pathological results, and all imaging modalities showed higher specificity values than sensitivity values in detecting metastatic LN. US demonstrated the best diagnostic performance in evaluating LN status, a finding that has been reported in previous studies [32-34]. Sensitivity and NPV were not satisfactory, however, despite US having the best results. Alvarez et al. [34] reported axillary US was moderately sensitive and quite specific in the diagnosis of axillary LN metastasis, and thus negative sonographic results do not exclude the presence of metastasis. US-guided biopsy, or aspiration of sonographically suspicious nodes, would be recommended to increase the specificity.

To our knowledge, the use of MRI with CAD for LN evaluation has not been reported. In this study, CAD showed limitations in identifying LN metastasis in invasive breast cancers. CAD had the worst correlation coefficient for agreement of determined LN status with pathological results among the breast imaging modalities studied. CAD also did not improve diagnostic performance values of breast MRI when compared with MRI alone in the evaluation of LN status. Therefore, CAD is useful in evaluating the breast itself, but may be limited in assessing LN status in breast cancer patients.

In terms of the detection of multifocality in invasive breast cancers, MMG, US, CT, and MRI had satisfactory results. FDG-PET had very low sensitivity (33.33%) even though it had high specificity (93.42%), and thus had an AUC value that was significantly lower than the other imaging modalities. Breast MRI had higher sensitivity (100%) and higher AUC value (0.809) than MMG, US, CT, or FDG-PET. CAD improved the specificity (77.63%) of breast MRI for detection of multiple breast cancers with preservation of the sensitivity of MRI, and thus had the best AUC value (0.888).

CAD for MRI has a number of advantages in detecting breast cancers. The system handles large images with speed and provides an angiomap and colored imaging that can correlate with kinetic features of a breast lesion. This enables easy detection of abnormal breast lesions. CAD also can improve lesion contrast and diagnostic performance, especially specificity [12]. These advantages may improve the ability to detect multifocality on MRI. Detection of mulfocality is essential to plan surgical methods and to reduce tumor recurrence. In this study, three patients who had multifocal cancers changed their surgical planning form conservation to mastectomy.

Although our preliminary results are promising, there were several limitations to the study. First, it is a retrospective review of nonconsecutive patients with invasive breast cancers. Second, the number of patients was small. Furthermore, we did not evaluate differences in the measurement of tumor extent or multifocality according to the tumor morphology on breast imaging. For example, mass or non-mass like enhancement on MRI may produce different results. Further validation using a larger study population is warranted. Third, tumor extents in each modality were not measured in the same orientation- e.g. we recorded tumor extent on mammography in either cranial or caudal orientation, on MRI in axial or sagittal orientation, on US in any orientation. Size measurement in these various orientations may affect the results, although we measured tumor extent in multiple planes and then recorded the longest diameter of a breast tumor in each modality. Fourth, the patients’ order was randomized. but the images were not presented to the radiologists in a randomized order. The radiologists independently evaluated a series of images from each patient, similar to the real clinical practice. However, this may produce a bias of image interpretation. It is recommended that a clinical study in a large population, utilize completely randomized image order to assess CAD utility. Fifth, we did not perform node-to-node correlation, but rather, case-to-case correlation with imaging and pathological results. Sixth, various CAD systems are customized for various kinds of MRI. Therefore, it is not certain that our results will be precisely reproducible if we use another kind of CAD or MRI. Finally, MR images were assessed in the light of mammographic or US findings.

## Conclusions

In conclusion, CAD for breast MRI can be a feasible method of evaluating tumor extent and multifocality in invasive breast cancer patients. Although manual measurement of tumor extent on MRI had a higher correlation coefficient with pathologic size than automatized measurement of CAD, there was no statistically significant difference. Detection of multifocality is important for surgical planning and prevention of residual tumors or recurrence. However, CAD is limited in evaluating LN status.US is the most accurate diagnostic tool for axillary LN metastasis and can help to overcome the limitations of CAD for MRI. Based on our preliminary results, utility of CAD may be extended from cancer detection to cancer staging, with particular regard to tumor extent and multifocality, for determination of treatment and prognosis in clinical settings.
